# Nutritional and Energy Profile of “No Added Sugar” Products Versus Their Conventional Counterparts on the Polish Food Market

**DOI:** 10.3390/nu17203266

**Published:** 2025-10-17

**Authors:** Aleksandra Kołodziejczyk, Justyna Nowak

**Affiliations:** 1 Faculty of Public Health in Bytom, Medical University of Silesia, 41-900 Bytom, Poland; 2 Department of Metabolic Disease Prevention, Faculty of Public Health in Bytom, Medical University of Silesia, 41-902 Bytom, Poland

**Keywords:** nutritional value, energy value, products without added sugar, sugar, sweeteners

## Abstract

**Background/Objectives**: The increasing presence of “no added sugar” products in the Polish food market provides consumers and nutritionists with access to products with varying nutritional compositions. Comparing the nutritional and energy values of products with and without added sugar provides objective data on their composition, which is important for informed diet planning and for monitoring differences between product groups. **Methods**: The research material included a total of 1278 food products, including 744 labeled “without added sugar” and 534 containing added sugar, obtained from four online stores and three offline retail outlets in Poland in the second and third quarters of 2023. The product assessment was based on an analysis of the nutritional and energy value, expressed per 100 g of each product. **Results**: The quantitative analysis revealed that products with added sugar were characterized by a higher energy value and a statistically significantly higher content of saturated fatty acids, carbohydrates, and sugars. **Conclusions**: Comparison of selected product groups revealed significant differences in nutritional and energy values. Analyzing these differences provides a practical overview of product composition and can be a useful source of information for consumers and nutritionists.

## 1. Introduction

In recent years, the prevalence of overweight and obesity has increased steadily. Current data from Poland indicate that 55.8% of adults are overweight, while 13.9% are obese [1]. High sugar consumption is one of the main factors contributing to the development of metabolic diseases [2,3]. In Poland, one in four adults consumes sweets at least twice a week [1], and the prevalence of diabetes is currently estimated at approximately 10% of the population [4]. The growing global burden of diabetes and obesity underscores the need for effective educational initiatives promoting optimal nutrition [2,3,5,6]. Sugar is a common dietary ingredient that provides high caloric value but contributes little to the overall nutritional quality of meals [6,7,8]. It is commonly found in foods such as desserts [9], milk and dairy products [9,10], beverages [11,12,13], cereal flakes [9,14], breath fresheners [15], snacks and sweets [9,11], as well as vegetables, fruits, and their products [9,11], and can significantly contribute to total sugar intake. In accordance with the definition set forth in Regulation (EC) No 1333/2008 of the European Parliament and of the Council, a product labeled as “with no added sugar” is defined as one to which no mono- or disaccharides or other substances used for their sweetening properties have been added during the manufacturing process [16]. The development of such products is an important part of food reformulation strategies aimed at reducing the adverse health effects associated with excessive sugar consumption [17].

Despite the absence of traditional sources of sugar, products containing sugar substitutes may still have a high energy value due to other ingredients in their composition. As a result, these products may not meet the expectations of individuals seeking to lose weight or maintain normal blood glucose levels [17]. Furthermore, excessive consumption of fats, especially saturated fatty acids, has been identified as a significant factor contributing to the development of obesity and other metabolic diseases [17,18,19]. A healthy lifestyle should include not only the regulation of dietary components with adverse effects but also the incorporation of regular physical activity [16,20,21,22,23].

Despite the growing number of products labeled as “no added sugar” [24,25,26], few studies have systematically compared their nutritional composition with that of products containing sugar. Most previous research has focused on the health effects of consuming sugar or sugar substitutes rather than providing a detailed analysis of the nutritional value of individual products. This leaves a gap in knowledge regarding their differences. Therefore, further research on products containing low-calorie sugar substitutes is needed to better understand their nutritional composition and potential dietary role.

The aim of this study was to quantitatively assess the nutritional and energy values of products labeled as “no added sugar” and their sugar-containing counterparts, sourced from selected online and offline stores in Poland. The results of this study may offer valuable information for consumers, nutritionists, and policymakers, supporting informed product choices and the development of health recommendations and strategies.

## 2. Materials and Methods

### 2.1. Materials

A total of 1278 food products, collected during the second and third quarters of 2023 from four online stores and three brick-and-mortar stores in Poland, were included in the analysis. Stores were selected at random, including both large retail chains and smaller local outlets, to obtain a cross-sectional sample that reflects the diversity of the retail market.

The primary inclusion criterion was a clear “no added sugar” claim on the product label. In the first stage, a market analysis was conducted to identify product groups available on the Polish market that met this criterion. Next, products containing added sugar were selected within the same food categories to enable cross-group comparisons. Whenever possible, products from the same manufacturer were selected, but this was not always possible. Therefore, the analysis included a comparison of nutritional values between groups of products with and without added sugar within the same food categories.

**Figure 1:**
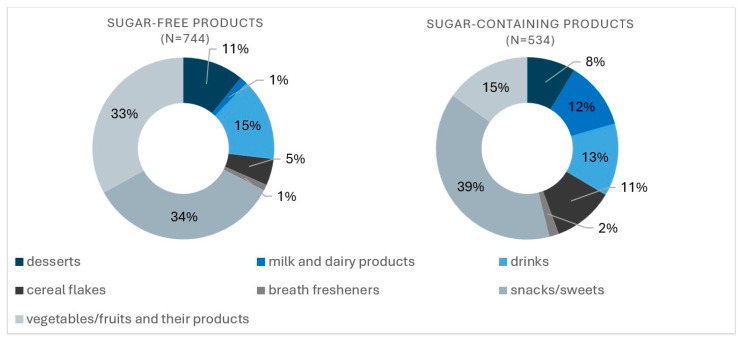
Percentage share of products in each category.

Ultimately, the analysis included 744 products labeled “no added sugar” and 534 products containing added sugar. The sample was classified into seven categories: desserts, milk and dairy products, drinks, cereal flakes, breath fresheners, snacks and sweets, and vegetables/fruits and their dairy products. Figure 1 presents the percentage of products in each category.

The analysis included a wide range of products representing different food groups. Table 1 presents a detailed breakdown of the products analyzed, including the number of products with and without added sugar within each food group and product type.

Due to European Union regulations not requiring the declaration of dietary fiber content [16], this parameter was analyzed only for products with available data. Consequently, fiber content information was obtained for 543 “no added sugar” products and 223 products containing sugar.

### 2.2. Methods

A database was created using Microsoft Excel 2019 (Microsoft Corporation, Redmond, WA, USA). To ensure the complete anonymity of the results and analyses, each product was assigned a unique numerical identifier. These identifiers were assigned randomly and had no influence on the analysis. The study did not require approval from a Bioethics Committee, as it was not a medical experiment. The research involved the analysis of information provided on the labels of “no added sugar” products and a comparison of these data with those of products containing added sugar.

The products collected for this study were subjected to a quantitative assessment. The assessment analyzed the protein, fat, and carbohydrate content in the quantity provided by 100 g or 100 m of a given product. Furthermore, the energy values of products designated as containing no added sugar were contrasted with their counterparts that were found to contain added sugar.

### 2.3. Data Processing

The resulting data were subjected to statistical analysis using the STATISTICA 13.3 program (StatSoft, Crakow, Poland). The data are presented using a number of statistical measures, including the arithmetic mean (M), standard deviation (SD), median (Me), first quartile (Q1), third quartile (Q3), minimum (Min), and maximum (Max). The distribution of the data was evaluated using the Shapiro–Wilk test. The data were subjected to statistical analysis using tests of significance to ascertain whether there were any differences between the groups. As most variables did not follow a normal distribution, the Mann–Whitney U test was employed for comparisons between two independent variables. In the analysis of the existence of interdependencies between variables, the Chi-squared test was utilized. In the statistical calculations performed, the level of significance α = 0.05 was applied, whereby results with a *p*-value less than 0.05 indicated the occurrence of significant differences between the variables.

## 3. Results

A quantitative analysis of the food products showed that the highest energy values were observed in the sweets and snacks group, while the lowest values were found in the beverage group. The median energy value for sweets and snacks without added sugar was 415.0 kcal/100 g (range: 348–518 kcal/100 g), whereas for sweets with added sugar, it was 495 kcal/100 g (range: 386–536 kcal/100 g). For beverages without added sugar, the median energy value was 35 kcal/100 g (range: 13–51 kcal/100 g), compared to 20 kcal/100 g (range: 19–29 kcal/100 g) for beverages with added sugar.

Significant differences (*p* < 0.05) in energy values were observed for cereals (*p* = 0.0001), breath freshening products (*p* = 0.0006), and sweets and snacks (*p* < 0.0001). No significant differences were found in the remaining product groups. These results are summarized in Table 2.

**Figure 2:**
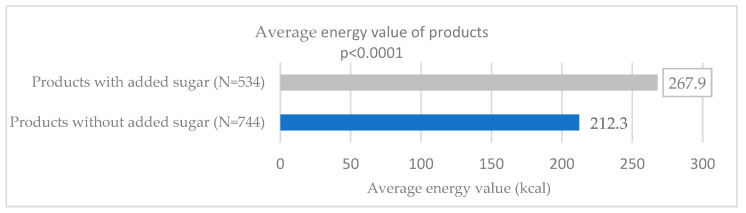
Comparison of the average energy value of the analyzed food products.

Comparison of the average energy values showed that products with added sugar contained 267.9 kcal/100 g, while products without added sugar contained 212.3 kcal/100 g. This difference was statistically significant (*p* < 0.0001). The results are presented in Figure 2.

Comparison of the average nutritional value of the products showed significant differences (*p* < 0.05) in the level of saturated fatty acids (*p* < 0.001), total carbohydrates (*p* < 0.0001), sugars (*p* < 0.0001), and fiber (*p* < 0.001) between products without and with added sugar. Products without added sugar were characterized by a higher median fiber content of 1.6 g/100 g of product (0.0–4.0 g/100 g of product), while products with added sugar had a higher median content of other nutritional values in the product. The highest fiber content in products without added sugar was 35 g/100 g of product, while in the case of products with added sugar, the maximum recorded value was 12 g/100 g of product.

The median total fat content was 1.2 g/100 g of the product without added sugar (0.2–12.0 g/100 g of the product) and 1.7 g/100 g of the product with added sugar (0.1–17.0 g/100 g of the product). Significant differences between groups for this macroelement were found only in the case of beverages, where the median for beverages with added sugar was 0.0, and for beverages without added sugar, 0.7. Table 3 presents detailed results in terms of total fat content in the analyzed groups.

The median content of saturated fatty acids was 0.2 g/100 g (0.0–2.7 g/100 g of the product) for products without added sugar and 0.8 g/100 g (0.0–5.7 g/100 g of the product) for products containing added sugar. In the group of products without added sugar, the content of saturated fatty acids ranged from 0 to 66.8 g/100 g of the product, while in products with added sugar, it ranged from 0 to 30.0 g/100 g of the product. Significant differences between groups were found only in the beverages and sweets, and snacks groups. Detailed results regarding saturated fatty acid content, broken down by product group, are presented in Table 4.

The median total carbohydrate content was 17.0 g/100 g of product without added sugar (10.0–49.8 g/100 g of product) and 42.4 g/100 g of product containing sugar (11.0–65.7 g/100 g of product). Comparison of carbohydrate content between groups revealed significant differences in carbohydrate content in the milk and dairy products, cereal flakes, and sweets and snacks groups. Each group showed a higher median value for products with added sugar. Detailed results are presented in Table 5.

Comparison of sugar content in products showed that in the case of products without added sugar, the median was 8.6 g/100 g of product (4.1–13.0 g/100 g of product), while in the case of products with added sugar, it was 22.6 g/100 g of product (9.6–43.0 g/100 g of product). Significant differences (*p* < 0.05) in sugar content were observed in all the analyzed product groups. Table 6 refers to the analysis of sugar content divided into the indicated product groups.

The overall analysis of fiber content in selected food products revealed no significant differences (*p* = 0.379) between products with and without added sugar. However, analysis by group revealed significantly higher median values for desserts, cereals, sweets and snacks, and vegetables, fruits, and their preserves. Detailed results are presented in Table 7.

The analysis showed no significant differences (*p* = 0.636) in terms of protein between products with and without added sugar in total. The highest protein value in 100 g of product, 55.7 g, was observed in the group of products without added sugar. The protein value in the case of products with added sugar ranged from 0 g/100 g to 24.0 g/100 g of product. Significantly higher median protein was found in products without added sugar, in the following groups: milk and dairy products, drinks, cereal flakes, breath fresheners, sweets and snacks, as well as vegetables, fruits, and their products.

The above results are presented in Table 8.

## 4. Discussion

Both physical and mental disorders, particularly poor nutritional status, may result from incorrect selection of the energy content of consumed foods and improper proportions of nutrients in the diet [27,28,29,30]. These factors may also play a key role in the pathogenesis of metabolic diseases. Therefore, it is important to consume foods with lower caloric content and an optimal nutritional composition [30,31,32,33]. Our analyses have shown that products with added sugar contain, on average, 55.6 kcal more per 100 g than their counterparts without added sugar. The highest average energy content (469.3 kcal/100 g) was observed in the sweets and snacks category, while the lowest (28.2 kcal/100 g) was found in the beverages category. This is supported by research conducted by Aleksandra Szydłowska and Danuta Kołożyn-Krajewska [34], who showed that products made from yeast dough without added sugar have a 15–25% lower caloric content compared to analogous products containing sugar. Our analyses also revealed a higher average fiber content in products without added sugar. These results are consistent with the observations of Szydłowska and Kołożyn-Krajewska [34], which resulted from the use of inulin as a sugar substitute in the traditional yeast dough recipe. Information focused on indicating the carbohydrate content in the tested products was consistent with the results of our own research, where a reduced amount of carbohydrates was observed compared to traditional products. Sugar present in products without added sugar came exclusively from natural raw materials and was not added in pure form. It is worth emphasizing that the increased amount of carbohydrates in these products was related to the high content of this ingredient in the recipe [34].

Our analyses have shown that products containing sugar are characterized by less favorable nutritional values. Products with added sugar exhibited statistically significantly higher energy content and higher levels of saturated fatty acids, carbohydrates, and sugar, as well as lower fiber content compared to products without added sugar. Similar results were obtained in the study by Maria Laura da Costa Louzada et al. [35] on ultra-processed foods. Their research found that analyzed products are characterized by higher energy density, higher sugar content, higher levels of saturated and trans fats, and lower fiber and potassium levels. Regarding energy value, it should be emphasized that diets with high energy density disrupt the body’s ability to regulate energy balance, which increases the risk of excessive weight gain [35,36,37,38]. Excessive sugar consumption significantly increases the risk of excessive weight gain, obesity, and tooth decay. Studies indicate that sugar is a leading cause of chronic diseases in the United States, including diabetes and cardiovascular disease [35,38]. The World Health Organization (WHO) recommendations for prevention, including reducing the risk of dental caries and obesity, emphasize the need to limit the consumption of free sugars to less than 10% of daily energy intake for children and adults [39]. Additionally, it is recommended to further reduce sugar consumption to less than 5% of daily energy intake, which may provide additional health benefits [2,39,40,41,42,43]. Highlighting the higher content of saturated fatty acids in products with added sugar, it is also important to note that excessive intake of these acids is associated with increased levels of low-density lipoprotein cholesterol (LDL), which in turn raises the risk of cardiovascular diseases and increases mortality [35,44,45,46,47,48]. To reduce this risk, it is recommended to include products with reduced saturated fatty acid content in the diet in order to limit their intake to less than 10% of total dietary energy [49,50,51].

In summary, it is worth noting that products without added sugar have more beneficial nutritional and energy values compared to their counterparts with added sugar. However, due to the variety of compositions of these products, an individual approach to the assessment of each product is important, taking into account all ingredients and potentially unfavorable substances. Research on the labeling of food products, conducted by Marta Sajdakowska et al. [52], showed that due to a lack of time, interest, and excess information in everyday life, consumers often skip reading product labels. This indicates the need for continuous expansion of knowledge and promotion of good practices in society in this area. Although products without added sugar may offer nutritional benefits compared to their sugared counterparts, they should not replace other elements of a balanced diet and should be consumed as part of a varied diet rather than excessively [39]. In this context, education on the consumption of highly processed food is extremely important, especially in connection with the growing problem of obesity. It is necessary to take action to constantly improve the daily diet and reduce health inequalities [53,54,55,56]. People with diabetes or on a reduced diet may be particularly interested in sweeteners, which are increasingly present in food products. In the context of guiding patients through the process of losing weight, it is important to consider whether recommending sugar substitutes to satisfy the need for a sweet taste will be beneficial for them.

## 5. Study Limitations

This study had several significant limitations that may affect the interpretation of the obtained results. Firstly, the scope of the sample was limited to selected online and stationary stores in Poland, which may not reflect the full spectrum of food products available on the market. Additionally, the analysis included only selected products, which limits the possibility of generalizing the results and their application to other groups of food products. In the case of some products, there may have been gaps in the data on nutritional value, which could have affected the accuracy of the assessment. Another limitation of the study is the dynamic nature of the food market, which could have affected the validity of the collected data—during the study, new products or changes in the composition of existing products could have appeared. The diversity of methods used by manufacturers to mark nutritional value is an additional difficulty, which may lead to inhomogeneity in comparing the analyzed products. Furthermore, the study did not take into account factors related to consumer preferences or motivations behind choosing products without added sugar, which could have affected the interpretation of the results in the context of consumer behavior and market trends. It is also important to note the potential selection bias resulting from including only products that declare fiber content. This nutrient is often only listed on labels when present in significant amounts or associated with a specific nutritional claim. Products with low fiber content or unfavorable for the manufacturer may be omitted, limiting the representativeness of the analyzed nutrient.

## 6. Conclusions

The quantitative assessment of products showed that products containing added sugar are characterized by a higher energy value and a higher content of saturated fatty acids and carbohydrates, which can lead to excessive calorie consumption and an increased risk of obesity and metabolic diseases in people who consume them. In contrast, products without added sugar were characterized by a favorable higher fiber content in most of the analyzed groups. Replacing products with added sugar with versions without added sugar is associated with a lower energy value and a changed macronutrient composition, which may affect the overall quality of the diet. However, it is important to increase consumer awareness to make informed dietary decisions. It is worth emphasizing that the analysis conducted concerned only the assessment of the nutritional and energy value of products. It would therefore be beneficial to increase public awareness of the benefits of reducing sugar consumption and promoting the habit of carefully reading food product labels. Effective consumer education could significantly contribute to improving dietary habits and the general health of the population, thus reducing health inequalities and supporting the prevention of metabolic diseases.

## Figures and Tables

**Table 1:** Number of analyzed products by food group and product type, distinguished by the presence or absence of added sugar.

Product Group	Product Type	No Added Sugar (N)	With Added Sugar (N)
Desserts	Flavors and Sauces	23	2
	Pudding	33	24
	Jelly	10	12
	Kissel	6	6
	Other Desserts	9	2
Milk and dairy products	Yogurt	7	26
	Dairy Drink	3	38
Drinks	Energy Drink	14	10
	Other Carbonated Drinks	16	29
	Plant-Based Drink	52	10
	Other Non-Carbonated Drinks	27	20
Cereal flakes	Muesli and granola	14	17
	Breakfast Cereals	21	41
Breath fresheners	Chewing Gum	2	-
	Lozenge	6	9
Sweets and snacks	Bar	80	40
	Candy	10	9
	Creams and Sweet Spreads	38	7
	Halva	13	9
	Savory Snacks	9	4
	Cookies	29	31
	Chocolate and Chocolate Products	32	59
	Cereal-Based Snacks	15	6
	Lollipop	1	7
	Filled Wafer Rolls	4	3
	Gummies	24	32
Vegetables. fruits and their products	Mousse/Paste	136	32
	Dried and Freeze-Dried Products	32	-
	Ketchup	4	22
	Fruit and Vegetable Drinks	74	27

**Table 2:** Comparison of the energy value in 100 g of the analyzed product groups.

Product Group	N	Energy Value (kcal) in 100 g of Product							
		M	SD	Min.	Max.	Me	Q1	Q3	*p*
Desserts	No added sugar (N = 81)	115.9	124.9	0.0	540.0	77.0	66.0	137.0	0.510
	With added sugar (N = 46)	98.1	98.9	46.0	507.0	74.5	50.0	90.0	
Milk and dairy products	No added sugar (N = 10)	65.9	14.6	41.0	79.0	71.0	58.0	77.0	0.068
	With added sugar (N = 64)	79.4	22.2	53.0	157.0	78.0	64.5	88.0	
Drinks	No added sugar (N = 109)	41.4	65.9	0.0	577.0	35.0	13.0	51.0	0.122
	With added sugar (N = 69)	28.2	18.4	10.0	142.0	20.0	19.0	29.0	
Cereal flakes	No added sugar (N = 35)	318.9	124.3	58.0	475.0	358.5	289.0	396.0	**0.0001**
	With added sugar (N = 58)	401.3	29.0	359.0	469.0	393.0	381.0	420.0	
Breath fresheners	No added sugar (N = 8)	218.9	42.2	149.0	244.0	241.0	195.5	242.5	**0.0006**
	With added sugar (N = 9)	391	4.4	383.0	399.0	392.0	389.0	392.0	
Sweets and snacks	No added sugar (N = 255)	416.0	129.3	0.0	710.0	415.0	348.0	518.0	**<0.0001**
	With added sugar (N = 207)	469.3	80.7	323.0	662.0	495.0	386.0	536.0	
Vegetables. fruits and their products	No added sugar (N = 246)	99.7	99.9	19.0	623.0	58.0	47.0	78.0	0.224
	With added sugar (N = 81)	93.5	55.0	18.0	168.0	107.0	20.0	142.0	

N—number of observations, M—arithmetic mean, SD—standard deviation, Me—median, Q1—first quartile, Q3—third quartile, Min.—minimum, Max.—maximum.

**Table 3:** Comparison of total fat content in 100 g of the analyzed product groups.

Product Group	N	Total Fat (g) in 100 g of Product							
		M	SD	Min.	Max.	Me	Q1	Q3	*p*
Desserts	No added sugar (N = 81)	3.0	8.4	0.0	44.0	1.5	0.0	1.9	0.126
	With added sugar (N = 46)	2.1	6.1	0.0	33.0	0.2	0.0	1.8	
Milk and dairy products	No added sugar (N = 10)	1.9	1.1	0.5	3.0	2.0	0.5	3.0	0.647
	With added sugar (N = 64)	2.0	1.8	0.0	8.4	1.6	0.8	2.2	
Drinks	No added sugar (N = 109)	1.4	5.1	0.0	52.0	0.7	0.0	1.5	**<0.0001**
	With added sugar (N = 69)	0.4	1.8	0.0	14.0	0.0	0.0	0.0	
Cereal flakes	No added sugar (N = 35)	7.6	6.2	0.3	23.0	5.7	2.3	12.7	0.997
	With added sugar (N = 58)	7.1	5.5	0.8	19.0	5.6	2.7	10.9	
Breath fresheners	No added sugar (N = 8)	0.5	0.4	0.0	0.9	0.6	0.2	0.8	0.312
	With added sugar (N = 9)	1.0	2.2	0.0	6.5	0.0	0.0	0.5	
Sweets and snacks	No added sugar (N = 255)	21.7	16.1	0.0	71.4	19.9	7.4	37.0	0.331
	With added sugar (N = 207)	20.5	14.0	0.0	55.0	24.0	7.1	31.0	
Vegetables, fruits, and their products	No added sugar (N = 246)	1.1	4.1	0.0	53.0	0.4	0.0	0.5	0.197
	With added sugar (N = 81)	0.3	0.3	0.0	0.9	0.5	0.0	0.5	

N—number of observations, M—arithmetic mean, SD—standard deviation, Me—median, Q1—first quartile, Q3—third quartile, Min.—minimum, Max.—maximum.

**Table 4:** Comparison of the content of saturated fatty acids in 100 g of the analyzed product groups.

Product Group	N	Saturated Fatty Acids (g) in 100 g of Product							
		M	SD	Min.	Max.	Me	Q1	Q3	*p*
Desserts	No added sugar (N = 81)	1.0	2.1	0.0	14.8	0.6	0.0	1.1	0.232
	With added sugar (N = 46)	0.8	1.8	0.0	8.7	0.1	0.0	1.1	
Milk and dairy products	No added sugar (N = 10)	1.1	0.8	0.3	2.0	0.7	0.3	2.0	0.512
	With added sugar (N = 64)	1.3	1.1	0.0	5.3	1.1	0.5	1.4	
Drinks	No added sugar (N = 109)	0.6	3.0	0.0	30.0	0.1	0.0	0.2	**<0.0001**
	With added sugar (N = 69)	0.1	0.2	0.0	1.6	0.0	0.0	0.0	
Cereal flakes	No added sugar (N = 35)	2.1	2.6	0.0	13.0	1.3	0.4	2.3	0.346
	With added sugar (N = 58)	2.2	2.1	0.1	11.0	1.6	0.7	3.4	
Breath fresheners	No added sugar (N = 8)	0.5	0.3	0.0	0.8	0.6	0.2	0.7	0.229
	With added sugar (N = 9)	0.6	1.4	0.0	4.0	0.0	0.0	0.0	
Sweets and snacks	No added sugar (N = 255)	7.8	8.2	0.0	66.8	5.6	1.6	13.0	**0.013**
	With added sugar (N = 207)	9.5	7.6	0.0	30.0	9.1	1.8	16.0	
Vegetables, fruits, and their products	No added sugar (N = 246)	0.2	0.6	0.0	7.4	0.0	0.0	0.1	0.622
	With added sugar (N = 81)	0.1	0.1	0.0	0.4	0.1	0.0	0.1	

N—number of observations, M—arithmetic mean, SD—standard deviation, Me—median, Q1—first quartile, Q3—third quartile, Min.—minimum, Max.—maximum.

**Table 5:** Comparison of total carbohydrate content in 100 g of the analyzed product groups.

Product Group	N	Total Carbohydrates (g) in 100 g of Product							
		M	SD	Min.	Max.	Me	Q1	Q3	*p*
Desserts	No added sugar (N = 81)	20.0	20.7	0.0	95.2	12.0	9.4	34.0	0.110
	With added sugar (N = 46)	17.2	13.6	1.8	71.1	14.5	11.0	16.0	
Milk and dairy products	No added sugar (N = 10)	6.3	2.8	3.2	13.9	5.9	5.0	6.1	**<0.0001**
	With added sugar (N = 64)	11.9	2.0	8.2	16.9	11.4	10.6	13.5	
Drinks	No added sugar (N = 109)	5.7	4.5	0.0	15.0	5.8	1.1	9.2	0.840
	With added sugar (N = 69)	5.6	2.6	1.2	12.0	4.7	4.4	6.8	
Cereal flakes	No added sugar (N = 35)	51.2	20.2	9.7	79.7	60.0	44.0	66.0	**<0.0001**
	With added sugar (N = 58)	72.7	7.8	53.0	86.0	73.6	65.7	80.0	
Breath fresheners	No added sugar (N = 8)	88.3	15.8	62.0	98.0	96.5	79.8	97.0	0.178
	With added sugar (N = 9)	95.3	5.7	81.0	99.0	98.0	96.0	98.0	
Sweets and snacks	No added sugar (N = 255)	49.1	20.1	3.5	98.2	48.0	36.0	61.0	**<0.0001**
	With added sugar (N = 207)	63.0	16.0	7.9	98.0	61.0	53.0	72.0	
Vegetables, fruits, and their products	No added sugar (N = 246)	20.9	18.9	3.0	86.0	13.3	11.0	20.0	0.120
	With added sugar (N = 81)	22.0	13.2	4.3	42.0	23.2	4.9	34.0	

N—number of observations, M—arithmetic mean, SD—standard deviation, Me—median, Q1—first quartile, Q3—third quartile, Min.—minimum, Max.—maximum.

**Table 6:** Comparison of sugar content in 100 g of the analyzed product groups.

Product Group	N	Sugars (g) in 100 g of Product							
		M	SD	Min.	Max.	Me	Q1	Q3	*p*
Desserts	No added sugar (N = 81)	4.5	5.0	0.0	35.5	4.3	0.9	7.0	**<0.0001**
	With added sugar (N = 46)	12.5	10.6	7.1	60.8	9.7	9.4	10.8	
Milk and dairy products	No added sugar (N = 10)	5.1	1.1	2.6	6.5	5.2	4.5	5.7	**<0.0001**
	With added sugar (N = 64)	11.3	1.9	7.9	15.0	11.1	9.9	12.7	
Drinks	No added sugar (N = 109)	3.9	3.3	0.0	13.0	4.1	0.3	6.0	**0.007**
	With added sugar (N = 69)	5.4	2.5	1.2	12.0	4.6	4.4	6.8	
Cereal flakes	No added sugar (N = 35)	6.7	4.6	0.1	17.2	6.9	2.0	9.0	**<0.0001**
	With added sugar (N = 58)	18.6	6.8	4.8	27.4	21.0	13.0	24.7	
Breath fresheners	No added sugar (N = 8)	0	0.0	0.0	0.0	0.0	0.0	0.0	**0.0006**
	With added sugar (N = 9)	73.9	11.6	61.0	99.0	73.0	68.0	80.0	
Sweets and snacks	No added sugar (N = 255)	21.0	20.4	0.0	77.9	11.0	4.6	38.8	**<0.0001**
	With added sugar (N = 207)	45.4	16.1	7.0	87.0	47.0	34.0	55.0	
Vegetables, fruits, and their products	No added sugar (N = 246)	16.1	16.2	0.0	73.1	10.8	9.0	13.0	**0.006**
	With added sugar (N = 81)	21.3	12.9	4.3	40.0	23.0	4.9	34.0	

N—number of observations, M—arithmetic mean, SD—standard deviation, Me—median, Q1—first quartile, Q3—third quartile, Min.—minimum, Max.—maximum.

**Table 7:** Comparison of total fiber content in 100 g of the analyzed product groups.

Product Group	N	Fiber (g) in 100 g of Product							
		M	SD	Min.	Max.	Me	Q1	Q3	*p*
Desserts	No added sugar (N = 58)	1.1	1.4	0.0	10.0	0.9	0.0	1.7	**0.001**
	With added sugar (N = 17)	0.2	0.3	0.0	1.3	0.1	0.0	0.1	
Drinks	No added sugar (N = 49)	1.7	5.9	0.0	35.0	0.3	0.0	0.6	0.2
	With added sugar (N = 14)	0.3	0.3	0.0	1.0	0.2	0.0	0.5	
Cereal flakes	No added sugar (N = 32)	8.5	4.9	0.8	19.0	8.4	4.3	11.0	**0.023**
	With added sugar (N = 58)	6.2	2.4	2.3	11.7	6.5	4.2	7.5	
Sweets and snacks	No added sugar (N = 198)	6.4	5.6	0.0	29.0	4.5	2.6	8.2	**<0.0001**
	With added sugar (N = 123)	3.7	2.5	0.0	12.0	3.3	2.0	5.3	
Vegetables, fruits, and their products	No added sugar (N = 206)	3.8	5.5	0.0	30.7	1.9	1.2	3.4	**0.009**
	With added sugar (N = 10)	1.2	0.6	0.5	2.8	1.1	1.0	1.1	

N—number of observations, M—arithmetic mean, SD—standard deviation, Me—median, Q1—first quartile, Q3—third quartile, Min.—minimum, Max.—maximum.

**Table 8:** Comparison of protein content in 100 g of the analyzed product groups.

Product Group	N	Protein (g) in 100 g of Product							
		M	SD	Min.	Max.	Me	Q1	Q3	*p*
Desserts	No added sugar (N = 81)	4.1	8.8	0.0	55.7	3.0	0.0	3.3	0.392
	With added sugar (N = 46)	2.1	1.7	0.0	7.7	1.7	1.3	3.1	
Milk and dairy products	No added sugar (N = 10)	4.5	2.9	1.9	12.4	4.0	3.1	4.4	**0.017**
	With added sugar (N = 64)	3.5	2.3	1.6	10.2	2.9	1.7	3.5	
Drinks	No added sugar (N = 109)	0.9	3.1	0.0	28.0	0.2	0.0	0.7	**<0.0001**
	With added sugar (N = 69)	0.3	1.0	0.0	6.0	0.0	0.0	0.0	
Cereal flakes	No added sugar (N = 35)	10.0	5.5	1.1	26.7	10.0	8.1	13.1	**0.003**
	With added sugar (N = 58)	8.4	1.5	5.3	12.8	8.5	7.6	9.0	
Breath fresheners	No added sugar (N = 8)	0	0.0	0.0	0.0	0.0	0.0	0.0	0.268
	With added sugar (N = 9)	0.2	0.4	0.0	1.0	0.0	0.0	0.1	
Sweets and snacks	No added sugar (N = 255)	9.1	7.1	0.0	45.0	7.4	4.3	12.3	**<0.0001**
	With added sugar (N = 207)	6.0	3.6	0.0	24.0	5.9	4.2	7.5	
Vegetables, fruits, and their products	No added sugar (N = 246)	1.5	2.6	0.0	21.7	0.6	0.5	1.2	**<0.0001**
	With added sugar (N = 81)	0.6	0.6	0.0	1.9	0.5	0.0	0.6	

N—number of observations, M—arithmetic mean, SD—standard deviation, Me—median, Q1—first quartile, Q3—third quartile, Min.—minimum, Max.—maximum.

## Data Availability

The data presented in this study are available on request from the corresponding author due to their large volume and complexity.

## Abbreviations

The following abbreviations are used in this manuscript:

M	Mean
SD	Standard deviation
Me	Median
Q1	First quartile
Q3	Third quartile
Min	Minimum
Max	Maximum
g	Gram
kcal	Kilocalories
